# Do pessimists report worse outcomes after total hip arthroplasty?

**DOI:** 10.1186/s12891-016-1045-4

**Published:** 2016-05-04

**Authors:** Jasvinder A. Singh, Robert C. Colligan, Megan M. O’Byrne, David G. Lewallen

**Affiliations:** Department of Orthopedic Surgery, Mayo Clinic, Rochester, MN USA; Department of Psychiatry and Psychology, Mayo Clinic, Rochester, MN USA; Department of Health Sciences Research, Mayo Clinic, Rochester, MN USA; Rheumatology Section, Medicine Service, VA Medical Center, Minneapolis, MN USA; Division of Rheumatology, Department of Medicine, University of Alabama at Birmingham (UAB), Birmingham, AL 35294 USA; University of Alabama, Faculty Office Tower, 805B, 510 20th Street S, Birmingham, AL 35294 USA

**Keywords:** Pessimism, Total hip arthroplasty, THA, Outcomes, Psychological risk factor, Pessimistic style

## Abstract

**Background:**

Seligman’s theory of causal attribution predicts that patients with a pessimistic explanatory style will have less favorable health outcomes. We investigated this hypothesis using self-reported hip pain and hip function 2- years after total hip arthroplasty (THA).

**Methods:**

Most THA patients had completed the Minnesota Multiphasic Personality Inventory (MMPI) during their usual clinical care long before THA (median, 14.7 to 16.6 years). Scores from the MMPI Optimism-Pessimism (PSM) scale were used to categorize patients as pessimistic (t-score >60) or non-pessimistic (t score ≤60). Outcomes were self-reported: (a) moderate-severe pain, (b) absence of “much better” improvement compared to preoperative hip function, and (c) moderate-severe activity limitation. Multivariable logistic regression was adjusted for gender, age and other covariates. Odds ratios (OR) with 95 % confidence intervals (CI) are presented.

**Results:**

We identified 507 patients with 565 primary THAs with an MMPI prior to primary THA, of whom 441 patients with 488 primary THAs had responded to hip pain and function follow-up surveys at 2-years post-surgery. Similarly, 202 patients with 235 revision THAs had an MMPI prior to surgery, of whom 172 patients with 196 revision THAs completed 2-year surveys. Among those with primary THA, pessimists reported (a) a non-significant trend toward more moderate-severe pain at 2-years with OR (95 % CI; *p*-value), 2.16 (0.90, 5.20; *p* = 0.08; reference, none-mild pain),; (b) no significant difference for absence of “much better” improvement in hip function at 2-years, 1.87 (0.77, 4.52; *p* = 0.16; reference, much better hip function); and (c) significantly higher rate of moderate-severe activity limitation at 2-years, 2.90 (1.25, 6.70; *p* = 0.01). Among revision THA cohort, pessimists reported no significant differences from non-pessimists in moderate-severe pain, improvement in hip function or moderate-severe functional limitation at 2-years.

**Conclusions:**

A pessimistic explanatory style was associated with moderate-severe activity limitation and a non-significant trend towards moderate-severe pain post-THA.

## Background

Total hip arthroplasty (THA) is a successful surgical procedure. THA is associated with significantly decreased pain, improved hip function, and better overall quality of life [1, 2]. Some patients have suboptimal pain and function outcomes following THA. A distressed psychological state around the time of surgery, can negatively impact outcomes after THA [3]. Psychological stressors can be broadly classified in two ways: first, as emotional *states* that fluctuate over time (e.g., depression, anxiety) and second, psychological *traits* that are relatively stable and chronic (such as a pessimistic explanatory style).

In our recent study of patients undergoing total knee arthroplasty (TKA), we found that a pessimistic explanatory style, determined years before the TKA, was associated with increased pain and decreased function at follow up, two years after surgery [4]. Studies over 30-years found that pessimistic explanatory style was significantly associated with increased mortality [5] and poorer self-reported quality of life [6] in general medical clinic cohorts. Thus, a pessimistic explanatory style, as a pre-existing trait, a psychological risk factor, can negatively impact important outcomes in medical and surgical patients. It is ***not*** known if certain, pre-existing, psychological characteristics, such as optimism or pessimism, affect outcomes after THA.

According to Seligman’s theory, people with pessimistic style attribute the causes of adverse events in their lives to themselves (i.e., an internal explanation, “It’s me…”), carry the expectation that the condition will persist (i.e., a stable explanation, “…happened again, as usual…”), and believe that it will affect other aspects of their life (a global explanation, “…and now… I’ll never get to..”). The converse characterizes an “optimistic” explanatory style [7–9]. Pessimistic style is associated with higher risks of poorer physical health, depression and lower academic accomplishments [10].

To our knowledge there are no studies that have determined if pessimistic explanatory style is associated with poorer outcomes after THA, except one study. The study found that optimism assessed on Life Orientation test immediately pre-THA was associated with short-term functional outcomes in the first 3 months after surgery [11]; however, the study did not adjust for depression, a factor associated with poor THA outcomes. The impact of pessimistic explanatory style on intermediate-term outcomes post-THA is not known. Conversely, it is not known if THA patients with poor outcomes differ in their explanatory styles assessed a decade or two prior to their surgery. In this research, our objectives were to assess whether pessimistic explanatory style is independently associated with poor outcomes of pain, activity limitations and the lack of improvement in hip joint function after primary or revision THA.

## Methods

### Study sample

The study cohort consisted of patients who had undergone primary or revision THA between 1993 and 2005 at Mayo Clinic in Rochester, MN, and completed the Mayo Hip Survey, a validated pain/function questionnaire with construct validity and reliability [12, 13], at 2-years post-surgery and a Minnesota Multiphasic Personality Inventory (MMPI) questionnaire at any time prior to their index THA. Surveys within six months either before or after the specific time point post-operatively were used for that time point. Patients with revision surgery prior to the 2-year mark were assessed prior to revision and that assessment was used as their 2-year survey. Patient-reported outcomes (PROs) of pain, function and activity limitation were derived from Mayo Hip Survey mailed to the patients or administered in the clinic during follow-up visits, as a part of regular clinical follow up. The questionnaire was administered on the telephone by trained Joint Registry staff to patients not responding to mailed survey and/or not returning for clinic follow-up. All MMPIs given at Mayo Clinic are stored electronically in a central database. The MMPI was given to patients as part of their routine care at Mayo Clinic, or as a result of participating in a research study; however, these THA patients were not specifically targeted to receive the MMPI. The overlap of the THA patients with follow-up Mayo Hip Surveys and the MMPI database comprised our sample. The Institutional Review Board at the Mayo Clinic approved the study, and waived the need for written informed consent for this study. We followed the Strengthening of Reporting in Observational studies in Epidemiology (STROBE) statement to describe this study [14].

### Predictor of interest: pessimism on MMPI profile

The original MMPI consisted of 550 unique true/false items pertaining to thoughts, feelings, attitudes, physical symptoms, emotional symptoms, and previous life experiences. The MMPI and its current revision, the MMPI-2 [15] are among the most widely used and thoroughly researched self-reported measures of personality assessment to date [16].

We used empirical approaches to assess the Optimism-Pessimism (PSM) scale of the MMPI [17], our main independent variable of interest. T-scores for the MMPI have been standardized to have a mean of 50 and standard deviation of 10. Recent studies have used t-scores above 60 (which is 50 plus one standard deviation) on a bi-directional scale of PSM using the MMPI item pool [18, 19] to indicate the pessimistic explanatory style. As previously [5, 6], we classified patients into two categories based on PSM scale scores preceding the THA as pessimists (T-score >60) or non-pessimists (T-score ≤60). MMPI scores have high internal consistency with Cronbach’s alpha of 0.85 for good events and 0.95 for bad events, reliability of 0.94 on linear composite reliability formula, and test-retest reliability of 0.90 [18]. MMPI scores have been shown to be stable with a correlation of 0.54 for negative events over 52 years [20].

### Outcomes of interest

We assessed three PROs, hip pain, improvement in hip function and activity limitation, as outcomes of interest. Index hip pain was assessed by a single question- “How much pain do you have in your hip that was operated?” Moderate or severe pain response were combined into moderate-severe pain and no or mild pain responses constituting the reference group.

Self reported improvement in hip function was assessed with a single question- “Compared to your condition before your hip surgery, how would you rate your hip function? We compared patients reporting “somewhat better, same or worse hip function” hip function with those in the reference category comprising “much better hip function”. The rationale for this categorization was that THA is an extremely successful surgery and most patients aim to achieve a much better hip function compared to preoperative status for this, mainly, elective procedure.

We categorized the responses to limitations in seven activities as follows: (1) walking, climbing stairs, sitting, rising from chair into ‘no’, ‘mild’, ‘moderate’ or ‘severe’ limitation for each activity; and (2) three activities including putting on shoes/socks, picking up objects from the floor, getting in/out of the car into none, moderate and severe limitation, due to no response category corresponding to mild limitation. Overall moderate-severe ADL limitation was defined as the moderate or severe limitation in ≥3 activities, as previously [21, 22].

### Covariates/confounders

Potential confounders i.e., gender and age (≤60, 61–70, >70 years) were included in the multivariable analyses of these PROs, due to their known previous associations with these outcomes in THA population [22–24]. In addition, multivariable analyses included other covariates, which could potentially impact pain/function outcomes as long as the number of events allowed inclusion of these variables (at least 10 events per variable): depression score (scale 2 (D) from the MMPI), comorbidity, median household income (a measure of socioeconomic level and an indirect measure of education level), preoperative pain and preoperative function.

### Statistical analyses

The association of pessimistic explanatory style with each of the three PROs was assessed in univariate analyses, separately in primary and revision THA cohorts, at 2-years. Multivariable logistic regression analyses adjusted for the potential confounders including age and gender. All multivariable-adjusted analyses used a generalized estimating equations (GEE) approach adjusting the standard errors for the within person correlation due to bilateral hip replacement and/or multiple operations on the same hip. Logistic regression analysis was used to determine which types of patients were more likely to have provided MMPI data, by comparing characteristics including age, gender, etc. Odds ratios (OR) with 95 % confidence intervals (CI) are reported, *p*-values <0.05 were considered significant, and *p*-values between 0.05 and 0.10 were considered a trend toward statistical significance. Analyses were performed using SAS 9.3 (Cary, NC) and Stata version 10 (College Station, TX).

## Results

### Demographics of the study cohort and non-response bias

We studied 488 patients with primary THA and 196 with revision THA who completed MMPIs and who had responded to post-THA pain and function-activity questionnaires at 2-years post-surgery. Median time from the time of MMPI completion to THA exceeded 14 years for both primary and revision THA cohorts (Table 1).

**Table 1:** Demographic and clinical characteristics of the study cohort

	Primary THA 2-year (*n* =488)		Revision THA 2-year (*n* =196)
Years from MMPI to surgery, median (range)	16.6 (0–41.3)		15.6 (0.2–40.9)
Age, Mean (SD)	67.4 (11.6)		67.8 (11.5)
%Men	42.6 %		46.9 %
Pessimists, *n* (%)	124 (25.4 %)		66 (33.7 %)
T-score for depression, Mean (SD)	56.8 (11.1)		59.3 (10.3)
Age groups, *n* (%)			
≤60 years	117 (24 %)		43 (21.9 %)
61–70 years	152 (31.1 %)		61 (31.1 %)
71+ years	219 (44.9 %)		92 (46.9 %)
Body Mass index (in kg/m^2^), *n* (%)			
≤25	120 (24.6 %)		53 (27 %)
> 25–29.9	186 (38.1 %)		77 (39.3 %)
30–34.9	114 (23.4 %)		51 (26 %)
35–39.9	42 (8.6 %)		6 (3.1 %)
≥40	21 (4.3 %)		6 (3.1 %)
Missing	5 (1 %)		3 (1.5 %)
ASA Score, *n* (%)			
Class I-II	268 (54.9 %)		90 (45.9 %)
Class III-IV	220 (45.1 %)		106 (54.1 %)
Charlson Index, Mean (SD)	1.6 (2.2)		1.4 (2.1)
Operative Diagnoses, *n* (%)		Operative Diagnoses, *n* (%)	
Rheumatoid Arthritis/ Other Inflammatory arthritis conditions	19 (3.9 %)	Loosening, Wear or Osteolysis	146 (74.5 %)
Osteoarthritis	409 (83.8 %)	Dislocation, Bone or Prosthesis Fracture, Instability, Non-Union	34 (17.3 %)
Avascular necrosis	48 (9.8 %)	Failed Arthroplasty with Components Removed or Infection	16 (8.2 %)
Other	12 (2.5 %)		
Cemented Implant, *n* (%)			N/A
Yes	64 (13.1 %)		
Hybrid	294 (60.2 %)		
No	130 (26.6 %)		

*N/A* Not applicable, *ASA* American Society of Anesthesiologists, *SD* standard deviation

For the primary THA 2-year follow-up cohort, mean age at surgery was 67.4 years, 43 % were men, 25 % had normal BMI of ≤25 and the underlying diagnosis was osteoarthritis in 84 % (Table 1). Characteristics of 2-year revision THA cohort were similar and are summarized in Table 1.

#### Patients with an MMPI

Patients in the primary 2-year follow up group were more likely to have had an MMPI if they were female, older, had higher comorbidity ASA class or household income, or lived closer to the medical center (Appendix 1). There were no significant effects of operative diagnosis or BMI. Revision THA 2-year cohort had similar characteristics related to likelihood of having MMPI (Appendix 2).

In unadjusted comparisons, pessimists did not differ from non-pessimists with regards to key demographic and clinical covariates in primary THA cohort (Table 2). Pessimists were more likely to be younger than non-pessimists in the revision THA cohort (Table 2).

**Table 2:** Univariate association of pessimism with selected patient characteristics

	Primary THA			Revision THA		
	Not Pessimists	Pessimists		Not Pessimists	Pessimists	
	No (*N* = 328)	Yes (*N* = 113)	*p* value	No (*N* = 112)	Yes (*N* = 60)	*p* value
Gender			0.23^a^			0.92^a^
Female	182 (55.5 %)	70 (61.9 %)		57 (50.9 %)	31 (51.7 %)	
Male	146 (44.5 %)	43 (38.1 %)		55 (49.1 %)	29 (48.3 %)	
Age Category			0.09^a^			0.04^a^
≤60	73 (22.3 %)	27 (23.9 %)		15 (13.4 %)	18 (30.0 %)	
61–70	93 (28.4 %)	43 (38.1 %)		34 (30.4 %)	18 (30.0 %)	
71–80	120 (36.6 %)	36 (31.9 %)		51 (45.5 %)	17 (28.3 %)	
>80	42 (12.8 %)	7 (6.2 %)		12 (10.7 %)	7 (11.7 %)	
BMI Category			0.91^a^			0.11^a^
<25	82 (25.2 %)	27 (24.3 %)		39 (35.8 %)	12 (20.0 %)	
25–29.9	129 (39.7 %)	42 (37.8 %)		39 (35.8 %)	25 (41.7 %)	
30–34.9	76 (23.4 %)	27 (24.3 %)		27 (24.8 %)	16 (26.7 %)	
35–39.9	27 (8.3 %)	9 (8.1 %)		2 (1.8 %)	4 (6.7 %)	
≥40	11 (3.4 %)	6 (5.4 %)		2 (1.8 %)	3 (5.0 %)	
ASA Score			0.38^a^			0.25^a^
1–2	187 (57.0 %)	59 (52.2 %)		55 (49.1 %)	24 (40.0 %)	
3–4	141 (43.0 %)	54 (47.8 %)		57 (50.9 %)	36 (60.0 %)	
Charlson Index			0.47^b^			0.20^b^
Mean (SD)	0.3 (0.4)	0.3 (0.5)		0.3 (0.5)	0.2 (0.3)	
Median (Q1, Q3)	0.2 (0, 0.4)	0.2 (0, 0.4)		0.2 (0, 0.4)	0.2 (0, 0.4)	
Income Category			0.23^a^			0.53^a^
<=$35 K	59 (21.1 %)	22 (23.9 %)		26 (28.6 %)	11 (22.4 %)	
>$35 K–$45 K	101 (36.2 %)	40 (43.5 %)		46 (50.5 %)	24 (49.0 %)	
>$45 K	119 (42.7 %)	30 (32.6 %)		19 (20.9 %)	14 (28.6 %)	
Distance Category			0.10^a^			0.91^a^
0–100 miles	178 (57.2 %)	61 (59.2 %)		44 (41.9 %)	23 (41.1 %)	
>100–500 miles	104 (33.4 %)	39 (37.9 %)		45 (42.9 %)	23 (41.1 %)	
>500 miles or Non-US	29 (9.3 %)	3 (2.9 %)		16 (15.2 %)	10 (17.9 %)	
Operative Diagnosis			0.11^a^			
Inflammatory Arthritis	12 (3.7 %)	1 (0.9 %)				
Osteoarthritis	278 (84.8 %)	95 (84.1 %)				
AVN	32 (9.8 %)	11 (9.7 %)				
Other	6 (1.8 %)	6 (5.3 %)				
Loosening/Wear or Osteolysis				89 (79.5 %)	39 (65.0 %)	0.08^a^
Dislocation, Bone or Prosthesis Fracture, Instability				17 (15.2 %)	13 (21.7 %)	
Failed arthroplasty with components removed or infection				6 (5.4 %)	8 (13.3 %)	
Cemented			0.83^a^			N/A
Yes	44 (13.4 %)	17 (15.0 %)				
Hybrid	199 (60.7 %)	65 (57.5 %)				
No	85 (25.9 %)	31 (27.4 %)				
Pre-operative Moderate/Severe Pain			0.58^a^			0.32^a^
No	9 (4.2 %)	2 (2.7 %)		14 (35.9 %)	6 (24.0 %)	
Yes	207 (95.8 %)	71 (97.3 %)		25 (64.1 %)	19 (76.0 %)	
Pre-operative 3+ Moderate/Severe Activity Limitations			0.22^a^			0.45^a^
No	14 (6.6 %)	2 (2.7 %)		9 (23.1 %)	4 (15.4 %)	
Yes	199 (93.4 %)	71 (97.3 %)		30 (76.9 %)	22 (84.6 %)	

^a^Chi-Square test

^b^Kruskal Wallis test

**Table 3:** Multivariable-adjusted association^a^ of Pessimism with outcomes 2-years after Primary THA

	n/N^b^ (%)	OR (95 % CI)	*p*-value
Moderate-Severe Pain vs. None-Mild Pain			
Pessimist			
No	27/339 = 8 %	1.0	
Yes	14/114 = 12.3 %	2.16 (0.90, 5.20) ^c^	0.08
Improvement in Hip Function to “Better/Same/Worse” vs. “Much Better”			
Pessimist			
No	41/323 = 12.7 %	1.0	
Yes	19/108 = 17.6 %	1.87 (0.77, 4.52) ^d^	0.16
Moderate-Severe Activity Limitation vs. None-Mild Activity Limitation			
Pessimist			
No	110/340 = 32.4 %	1.0	
Yes	49/116 = 42.2 %	**2.90 (1.25, 6.70)** ^**e**^	**0.01**

Significant odds ratios are in bold; Numbers rounded to the nearest digit for percentages

^a^ Multivariable logistic regression models adjusted for covariates consistent with 1 variable per 10 events rule. Variables were added in the following order due to clinical relevance: gender and age; depression; medical comorbidity; income; preoperative pain and function

^b^ Totals may not add up exactly to 488 at 2-years due to missing responses to each of the variable

^c^ Model adjusted for age, sex, depression score (41 events)

^d^ Model adjusted for age, sex, depression score and Charlson index, since adding income prevented the model from converging (60 events)

^e^ Model adjusted for age, sex, depression score, Charlson index, income, preoperative pain and preoperative function (86 events)

### Pessimistic explanatory style and outcomes 2-years after primary THA

12.3 % of pessimists versus 8.6 % of non-pessimists had moderate-severe pain 2-years after primary THA leading to an odds ratio of 2.16 (95 % CI: 0.90, 5.20; *p* = 0.08) in pessimists compared to non-pessimists in the multivariable-adjusted logistic model (Table 3). 17.6 % pessimists versus 12.7 % non-pessimists reported lack of improvement of hip function to much better at 2-year follow up. Multivariable-adjusted odds of lack of much better hip function improvement at 2-years were higher in pessimists, but not statistically significant, OR was 1.87 (95 % CI: 0.77, 4.52; *p* = 0.16). 42.2 % pessimists versus 32.4 % non-pessimists reported moderate-severe activity limitation 2-years after primary THA, which was statistically significant, OR was 2.90 (95 % CI, 1.25, 6.70; *p* = 0.01) (Table 3). Even when using an adjusted *p*-value threshold for multiple comparisons (three outcomes in this cohort, adjusted *p*-value of 0.017), this was statistically significant.

### Pessimistic explanatory style and outcomes 2-years after revision THA

Pessimists were not significantly different than non-pessimists to report moderate-severe pain, lack of “much better” improvement in hip function or moderate-severe activity limitation 2 years post-revision THA, ORs were 1.18 (95 % CI: 0.50, 2.81; *p* = 0.71), 1.08 (95 % CI: 0.52, 2.27; *p* = 0.83) and 1.73 (95 % CI: 0.76, 3.94; *p* = 0.19), respectively (Table 4).

**Table 4:** Multivariable-adjusted association^a^ of Pessimism with outcomes 2-years after Revision THA

	n/N^b^ (%)	OR (95 % CI)	*p*-value
Moderate-Severe Pain vs. None-Mild Pain			
Pessimist			
No	28/123 = 22.8 %		
Yes	17/66 = 25.8 %	1.18 (0.50, 2.81) ^c^	0.71
Improvement in Hip Function to “Better/Same/Worse” vs. “Much Better”			
Pessimist			
No	38/110 = 34.5 %	1.0	
Yes	22/59 = 37.3 %	1.08 (0.52, 2.27) ^d^	0.83
Moderate-Severe Activity Limitation vs. None-Mild Activity Limitation			
Pessimist			
No	67/123 = 54.5 %	1.0	
Yes	42/63 = 66.7 %	1.73 (0.76, 3.94) ^e^	0.19

^a^ Multivariable logistic regression models adjusted for covariates consistent with 1 variable per 10 events rule. Variables were added in the following order due to clinical relevance: gender and age; depression; medical comorbidity; income; preoperative pain and function

^b^ Totals may not add up exactly to 196 at 2-years due to missing responses to each of the variable

^c^ Model adjusted for age, sex, depression score (45 events)

^d^ Model adjusted for age, sex, depression score and Charlson index; adding income prevents the model from converging (60 events)

^e^ Model adjusted for age, sex, depression score, Charlson index, and income (85 events; no other variables had univariate *p*-values less than 0.2)

## Discussion

To our knowledge, this is the first study to examine the association between the preexisting psychological trait of a pessimistic explanatory style and PROs at intermediate post-operative follow-up in patients undergoing THA. We investigated intermediate-term PROs of self-reported pain, activity limitation and the lack of much better improvement in hip joint function. It is important to note that scores on the PSM scale used to classify our patients were obtained from MMPIs that had been completed a median of 16–17 years before their primary THA. The MMPI scores are valid, reliable and stable across several years [18, 20]. Specifically, previous studies showed that MMPI scores have high internal consistency, test-retest reliability [18] and stability over 52 years [20]. Several study findings deserve further discussion and have important implications.

Our study found that pessimism was associated with statistically significantly higher odds of moderate-severe activity limitation at 2-years in primary THA, a non-significant trend for moderate-severe pain and no statistically significant association with the lack of improvement in hip function to “much better” at 2-years post-primary THA and, with respective odds of 2.90, 2.16 and 1.87 (*p* = 0.01, 0.08 and 0.16 respectively). The striking similarity of these odd ratios to those noted previously in a primary TKA cohort, and the consistency of associations across univariate and multivariable-adjusted models, is supportive of these associations being real. Several reasons might explain poorer outcomes in pessimists: (1) Pessimists may be less resilient in adapting to the challenges of major surgery such as THA and this may impact their ability to successfully perform physical rehabilitation, as noted in patients with depression [25, 26], and ultimately their functional outcome; (2) pessimistic explanatory style may have had a negative influence on outcome expectation and lower patient expectation is associated with worse results and lower patient satisfaction after arthroplasty [27–31].

Our study findings have several important implications. First, this study identifies and informs patients and surgeons that pessimistic style is a novel risk factor for poor functional outcome after primary THA, in addition to previous known factors such as demographics (age, gender) and depression [22, 23]. Screening for pessimism in patients with other risk factors for poor outcomes may be indicated. Second, this knowledge can allow the surgeon and patients to have realistic expectations of outcomes following primary THA. Third, a cognitive-behavioral intervention may be appropriate to improve outcomes in patients with pessimistic explanatory style undergoing primary THA; this remains to be tested. These findings also add to the recent findings that pessimism is associated with poorer self-reported health in general medical [6], and cancer patients [32, 33], the success of smoking cessation among medical patients [34] and with mortality in medical outpatients [5] and in a college sample [35].

Pessimistic style was associated with numerically higher odds of moderate-severe pain, but this did not reach statistical significance in multivariable-adjusted models. The odds of moderate-severe pain of 2.16 (95 % CI, 0.90, 5.20; *p* = 0.08) between pessimism and moderate-severe pain 2-years after primary THA in the present study is similar to the previously reported statistically significant odds ratio of 2.21 (95 % CI, 1.12, 4.35; *p* = 0.02) for pessimism and moderate-severe pain 2-years after primary TKA cohort [4]. Importantly, the direction and magnitude of association is similar to that noted in the previous study for a TKS cohort. It is possible that the association of pessimism with moderate-severe pain may be weaker after THA vs. TKA; however, the similarity of odds ratio indicates this was likely not the case. A more likely explanation is that the association in primary THA failed to reach statistical significance due to a smaller sample size in the present study, with our current study consisted of 488 patients with only 41 patients with reporting moderate-severe pain (outcome of interest) in our current THA study versus compared to 783 patients with 87 patients with reporting moderate-severe pain (outcome of interest) in the previous TKA study [4].

We noted that the magnitude of associations of pessimistic style with poorer functional and pain outcomes in patients undergoing revision THA (OR ranging 1.08 to 1.73) was much weaker to non-existent. This finding indicates that the pathways to pain and function outcomes after revision THA are likely different than those after primary THA. The lack of significance may at least partially be due to a smaller sample size than primary THA (202 vs. 507 patients).

These study findings must be interpreted considering study limitations. First, the patients chosen by the physicians and asked to complete an MMPI were not chosen at random. However, this bias mirrors clinical practice, which improves generalizability of findings, where only patients suspected to have psychological distress would be referred for such an evaluations. This tool can’t and shouldn’t be administered to all-comers for THA, due to the length of the survey and the low prevalence of pessimistic explanatory style. Explanatory style is unlikely to have biased completion and return rates for the MMPIs among the medical outpatients included in our study, as reported previously [36]. Our study was completed entirely with the original version of the MMPI. For clinicians or researchers interested in replicating or disputing our findings, a revised version of the PSM scale, the PSM-R [19] has been developed for the MMPI-2, the current version of the original MMPI. Less than 1 % THA were bilateral, and we used GEE to account for correlated observations. Residual confounding due to other known and unknown variables is another potential limitation given that the observational study design. All study outcomes were PROs that are self-reported. However PROs are widely accepted as the most important outcomes after an elective surgery such as THA. The long-time lag between MMPI profile and THA does not threaten the validity of these findings, since Seligman’s theory postulates pessimism to be an enduring trait, one that has been validated among medical outpatients over a 30-year follow up [5, 6].

Some of the patients studied underwent THA 20 years ago, which should be considered while interpreting findings. However, while some changes in surgical techniques or implants have occurred, we are not aware of any data demonstrating strong upwards or downwards trends in patient-reported outcomes in the last two decades. Therefore, the findings presented here are likely relevant to current THA patient populations. We used a single-item pain question rather than a multi-item questionnaire such as Western Ontario McMaster Osteoarthritis Index (WOMAC) or Knee Osteoarthritis Outcome Score (KOOS). Potential disadvantage of using a single-item are that longer instruments (WOMAC, KOOS) are more validated. Advantages of a single-item pain assessment are the feasibility of administering a single-item in a busy clinical practice or even in research settings, which likely improves response rate and generalizability of findings. Pain as the fifth vital sign has always been assessed as a single question in routine clinical settings in the U.S. for years, not with multi-item scales. In addition, the summated scores may not always superior to a single item assessment scale [37].

Our study has several strengths. Study sample size was relatively large, validated patient-relevant outcomes were used, both primary and revision THA populations were studied and we performed multivariable-adjusted analyses including important covariates.

## Conclusions

In conclusion, we found that a pessimistic explanatory style before surgery was associated with significantly less favorable improvement in function/ activity limitation after primary THA and possibly higher risk of moderate-severe pain. Screening for patients suspected to be at risk (pessimists) may be indicated, based on clinical suspicion. In those with known pessimistic explanatory style, screening for other risk factors for poorer PRO outcomes post-primary THA may be indicated. Psycho-educational and cognitive- behavioral strategies may be evaluated for pessimists undergoing elective primary THA.

### Data availability

These data will be made available to other researchers upon request, after meeting appropriate data privacy and Human subject protection requirements, as per institutional requirements.

## Appendix

**Table 5:** Responders to MMPI survey 2-year follow-up for Primary THA

Total Obs	Yes Events	Variable	Level	Yes Events/Trials	Odds Ratio	95% CI for Odds Ratio	*p*-value
5707	488	Gender	Female	280/2929 = 9.6%			
			Male	208/2778 = 7.5%	0.77	(0.62,0.94)	0.01
5707	488	Age Category	≤60	117/1730 = 6.8%			
			61–70	152/1759 = 8.6%	1.30	(0.99,1.72)	0.06
			71–80	169/1721 = 9.8%	1.50	(1.15,1.97)	<0.01
			>80	50/497 = 10.1%	1.54	(1.07,2.23)	0.02
5679	483	BMI Category	<25	120/1387 = 8.7%			
			25–29.9	186/2224 = 8.4%	0.96	(0.75,1.24)	0.78
			30–34.9	114/1361 = 8.4%	0.97	(0.72,1.29)	0.81
			35–39.9	42/479 = 8.8%	1.01	(0.68,1.51)	0.94
			≥40	21/228 = 9.2%	1.07	(0.62,1.85)	0.81
5678	488	ASA Score	1–2	268/3540 = 7.6%			
			3–4	220/2138 = 10.3%	1.40	(1.16,1.70)	<0.01
5701	488	Charlson Index (5 point increase)	N/A	N/A	1.76	(1.44,2.14)	<0.01
4792	412	Income Category	>$45K	167/1507 = 11.1%			
			≤$35K	91/1234 = 7.4%	0.64	(0.48,0.85)	<0.01
			>$35K–$45K	154/2051 = 7.5%	0.65	(0.51,0.83)	<0.01
5511	459	Distance Category	0–100 miles	266/2664 = 10%			
			>100–500 miles	155/2280 = 6.8%	0.66	(0.53,0.82)	<0.01
			>500 miles or Non-US	38/567 = 6.7%	0.65	(0.44,0.95)	0.03
5707	488	Operative Diagnosis	Inflammatory Arthritis	19/148 = 12.8%			
			AVN	48/409 = 11.7%	0.90	(0.47,1.73)	0.76
			Osteoarthritis	409/4944 = 8.3%	0.61	(0.34,1.09)	0.09
			Other	12/206 = 5.8%	0.42	(0.19,0.95)	0.04

**Table 6:** Responders to MMPI survey 2-year follow-up for Revision THA

Total Obs	Yes Events	Variable	Level	Yes Events/Trials	Odds Ratio	95% CI for Odds Ratio	*p*-value
2687	196	Gender	Female	104/1437 = 7.2%			
			Male	92/1250 = 7.4%	1.02	(0.74,1.41)	0.91
2687	196	Age Category	≤60	43/796 = 5.4%			
			61–70	61/731 = 8.3%	1.59	(1.03,2.48)	0.04
			71–80	71/902 = 7.9%	1.50	(0.97,2.32)	0.07
			>80	21/258 = 8.1%	1.55	(0.86,2.79)	0.14
2652	193	BMI Category	<25	53/777 = 6.8%			
			25–29.9	77/1024 = 7.5%	1.11	(0.77,1.61)	0.58
			30–34.9	51/576 = 8.9%	1.33	(0.87,2.04)	0.19
			35–39.9	6/183 = 3.3%	0.46	(0.20,1.10)	0.08
			≥40	6/92 = 6.5%	0.95	(0.35,2.57)	0.92
2679	196	ASA Score	1–2	90/1384 = 6.5%			
			3–4	106/1295 = 8.2%	1.28	(0.95,1.73)	0.10
2682	196	Charlson Index (5 point increase)	N/A	N/A	2.23	(1.60,3.11)	<0.01
2049	157	Income Category	>$45K	37/480 = 7.7%			
			≤$35K	41/604 = 6.8%	0.87	(0.54,1.41)	0.58
			>$35K–$45K	79/965 = 8.2%	1.07	(0.70,1.63)	0.76
2575	184	Distance Category	0–100 miles	77/829 = 9.3%			
			>100–500 miles	77/1426 = 5.4%	0.56	(0.39,0.80)	<0.01
			>500 miles or Non-US	30/320 = 9.4%	1.01	(0.62,1.65)	0.97
2687	196	Operative Diagnosis	Loosening/Wear or Osteolysis	146/1959 = 7.5%			
			Dislocation, Bone or Prosthesis Fracture, Instability, Non-Union	34/447 = 7.6%	1.02	(0.69,1.52)	0.91
			Failed Prior Arthroplasty with Components Removed or Infection	16/281 = 5.7%	0.75	(0.43,1.32)	0.32

## Abbreviations

ASA: American society of anesthesiology
BMI: Body mass index
CI: confidence interval
MMPI: Minnesota multiphasic personality inventory
OR: odds ratio
PROs: patient-reported outcomes
PSM: Optimism-pessimism scale
THA: total hip arthroplasty
